# A Simplified, Sensitive Phagocytic Assay for Malaria Cultures Facilitated by Flow Cytometry of Differentially-Stained Cell Populations

**DOI:** 10.1371/journal.pone.0038523

**Published:** 2012-06-04

**Authors:** Chuu Ling Chan, Laurent Rénia, Kevin S. W. Tan

**Affiliations:** 1 Laboratory of Molecular and Cellular Parasitology, Department of Microbiology, Yong Loo Lin School of Medicine, National University of Singapore, Singapore, Singapore; 2 Laboratory of Malaria Immunobiology, Singapore Immunology Network, Immunos, Singapore, Singapore; State University of Campinas, Brazil

## Abstract

**Background:**

Phagocytosis of infected and uninfected erythrocytes is an important feature of malaria infections. Flow cytometry is a useful tool for studying phagocytic uptake of malaria-infected erythrocytes *in vitro*. However, current approaches are limited by the inability to discriminate between infected and uninfected erythrocytes and a failure to stain the early developmental ring stages of infected erythrocytes. The majority of infected erythrocytes in circulation are of the ring stage and these are therefore important targets to study.

**Methodology/Principal Findings:**

*In vitro P. falciparum* cultures comprising infected and uninfected erythrocytes were labeled and exposed to cells derived from the human monocytic THP-1 cell line. Phagocytosis was assayed by flow cytometry. Dual labeling of *Plasmodium* DNA and erythrocyte cytoplasm with dihydroethidium and CellTrace™ Violet respectively allowed, for the first time, the detection and enumeration of phagocytes with ingested erythrocytes from both early ring- and late schizont-stage *P, falciparum* cultures. The sensitivity of the method was tested using varying conditions including phagocyte type (monocytes versus macrophages), parasite stage (rings versus schizonts), and negative (incubation with cytochalasin D) and positive (incubation with immune sera) effectors of phagocytosis. The current assay clearly demonstrated uptake of infected and uninfected erythrocytes exposed to phagocytes; the extent of which was dependent on the conditions mentioned.

**Conclusions:**

We describe a simple, sensitive and rapid method for quantifying phagocytosis of *P. falciparum*-infected erythrocytes, by flow cytometry. This approach can be applied for studying parasite-phagocyte interactions under a variety of conditions. The investigation of phagocytosis of *P. falciparum*-infected erythrocytes can extend from looking solely at late-staged infected erythrocytes to include early-staged ones as well. It does away with the need to purify infected cells, allowing the study of effects on neighboring uninfected cells. This method may also be translated for use with different types of phagocytes.

## Introduction

Malaria is one of the most prevalent epidemic diseases in the world, particularly in the subtropical and tropical regions, with 300 to 500 million new infections and approximately 1 to 2 million deaths annually [1]. The control of this disease is hindered by spreading resistance of the malaria parasite, *Plasmodium* species, to common antimalarials such as the quinolines, the antifolates. Recent discovery of resistance to artemisin-derivatives has urged research to search for new therapeutic initiatives [2]. One of the keys in the fight against the disease is a clear understanding of the mechanisms of *Plasmodium* immune evasion, the host immune response and the corresponding pathogenicity. The host immune response during a malaria infection involves both innate immunity and adaptive immunity. Innate immunity is important in controlling parasitemia in the acute phase of infection and for initiating adaptive immunity. Specific antibodies produced against *Plasmodium* are involved in elimination and resolution of the chronic phase of the malaria infection [3]. Phagocytosis of infected red blood cells represents the first line of defense against the parasite.

Understanding how phagocytosis of malaria-infected erythrocytes (iRBCs) and subsequent antigen presentation facilitates the formation of specific antibodies for parasite clearance is important in the development of new strategies for treating malaria. The parasites encounter phagocytes at different points throughout its life cycle. For example, after being injected into the host by the mosquito vector, sporozoites need to evade monocytes/macrophages in the skin dermis to enter the blood stream [4]. In the liver, sporozoites elude Kupffer cells in the liver sinusoids to reach their target hepatocytes [4]. Upon entering the erythrocytic cycle, iRBCs become increasingly antigenic as *Plasmodium* develops and inserts its antigens on the cell surface of erythrocytes [5]. As these iRBCs circulate in the blood, they come into contact with and are recognized by monocytes, neutrophils, dendritic cells and tissue macrophages. After ingestion by phagocytes, parasite antigens are processed and presented to T cells, either directly or indirectly, for the initiation of adaptive immunity [6], [7], [8].

Great research emphasis has been placed on the erythrocytic cycle where malaria pathology manifests. However, investigating phagocytosis in the erythrocytic cycle is complicated by the presence of two populations of cells: iRBCs and uninfected erythrocytes (uRBCs). Traditionally, microscopy has been used to enumerate cells that have been engulfed [9], [10], [11]. While this method permits discrimination between phagocytosis of an infected erythrocyte and an uninfected erythrocyte and allows the number of erythrocytes taken up per phagocyte to be counted, it is extremely time-consuming, labour-intensive and susceptible to human error.

The preferential use of flow cytometry in phagocytic assays has become increasingly evident due to its ability to acquire large amounts of data in a short period of time. Such rapid data acquisition is objective and permits analysis of thousands of cells per sample, leading to smaller errors. But *Plasmodium* is an intracellular parasite and phagocytosis of iRBCs and uRBCs cannot be readily distinguished with current staining methods. The purification of iRBCs prior to staining can be used to circumvent this problem [12], [13], [14], [15]. However, iRBC isolation is not physiological and the effects of iRBCs on neighboring uRBCs are neglected.

A method previously described by Tippett *et al* [16] employed ethidium bromide (EB) and fluorescein isothiocyanate (FITC) to label parasites of the later trophozoite stage and erythrocytes, respectively. However, we have observed that EB was unable to stain earlier ring-staged iRBCs which is important when investigating phagocytosis as majority of iRBCs in circulation are of the early ring-stage. While many phagocytic studies focus on the later trophozoite- and schizont-staged iRBCs, ring-staged iRBCs have also been demonstrated to be detected by macrophages and possibly play a role in innate immune control [17]. It is thus necessary to study the interaction between phagocytes and iRBCs of various developmental stages, including those of ring stages.

Here, we propose a method to differentially stain iRBCs from uRBCs which eliminates the need for iRBC purification and allows the study of phagocytosis in a system that closely resembles *in vivo* conditions. Using a combination of dihydroethidium (DHE) and CellTrace™ Violet (Violet) to label the parasite DNA and erythrocyte cytoplasm respectively, allows the uptake level of uRBCs, early ring- and late schizont-staged 3D7 *Plasmodium falciparum* cultures by THP-1 phagocytes to be measured.

## Results

### Dihydroethidium Staining and Comparison with Ethidium Bromide

**Figure 1:**
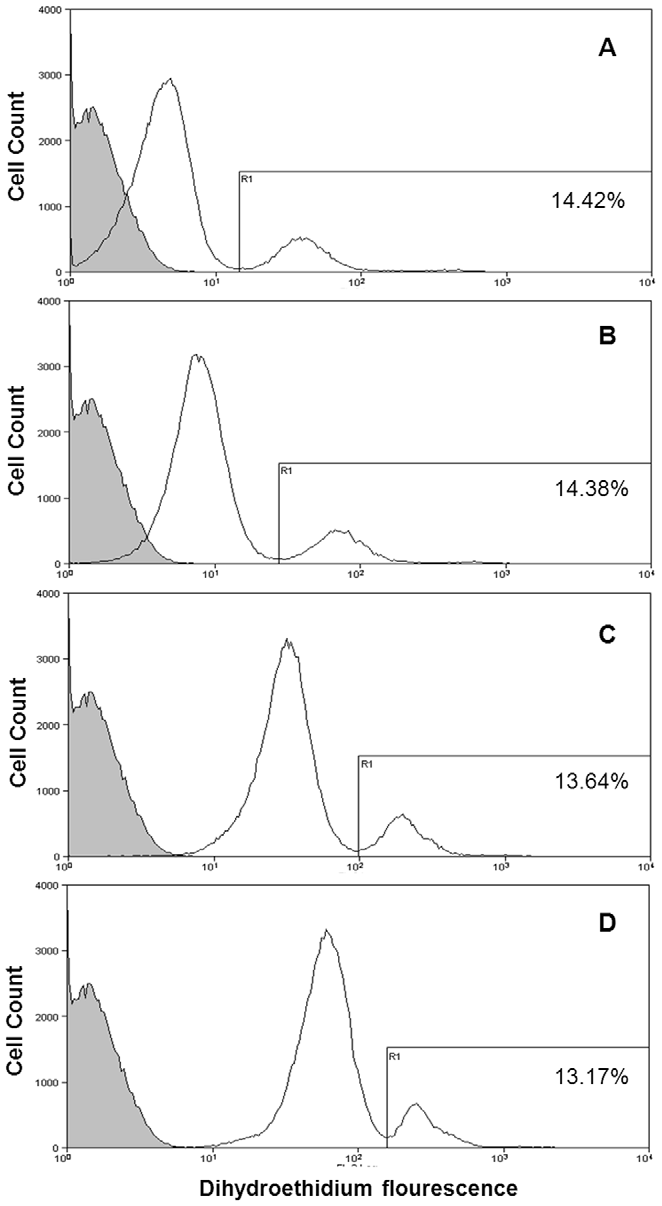
Ring-staged 3D7 *Plasmodium falciparum* culture labeled with varying concentrations of dihydroethidium. Ring-staged cultures at about 15% parasitemia, 1% hematocrit were labeled with **A**) 5 µg/ml, **B**) 10 µg/ml, **C**) 25 µg/ml and **D**) 50 µg/ml DHE for 20 min at 37°C and each was compared to an (gray shade histogram) unstained blood control using flow cytometry. Cultures labeled with 5 µg/ml DHE showed a clear resolution between infected and uninfected erythrocytes (obtaining a parasitemia of 14.42% which corresponded to that in the Giemsa blood smear) and a low uRBC background as compared to the other concentrations.

**Figure 2:**
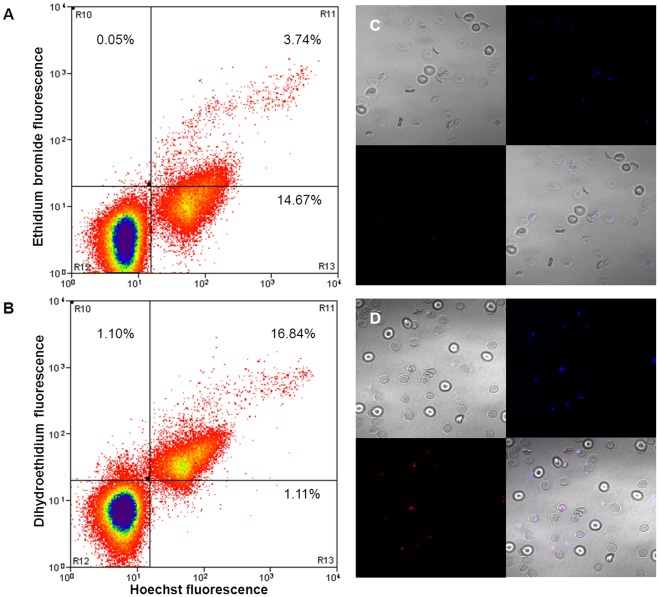
Comparison between EB and DHE labeling, using Hoechst 33342 as a comparator. Ring-staged *Plasmodium falciparum* cultures at 20% parasitemia, 1% hematocrit were labeled with (**A, C**) 10 µg/ml EB or (**B, D**) 5 µg/ml DHE, both of which were co-stained with 1 µg/ml Hoechst 33342, for 20 min at 37°C before analysis using flow cytometry and confocal imaging. **A**) Only about 20% of Hoechst-labeled iRBCs were stained with EB whereas **B**) more than 90% of Hoechst-labeled iRBCs were stained with DHE.

To determine the optimal concentration for DHE-labeling of infected erythrocytes, concentrations of 5 µg/ml, 10 µg/ml, 25 µg/ml and 50 µg/ml were used to stain ring-staged *Plasmodium falciparum* culture of about 15% parasitemia. At 5 µg/ml DHE, the resolution between the parasite-infected population and the uninfected population was the most distinct, with the uninfected population having a relatively low background, as compared to 10 µg/ml, 25 µg/ml and 50 µg/ml DHE (Figure 1). In addition, the parasitemia obtained via flow cytometry analysis at 5 µg DHE per milliliter was 14.42% which corresponded to the Giemsa smear (Figure1A and Figure S1).Comparing DHE with two other commonly used DNA stains (EB and Hoechst 33342), ring-staged parasite cultures were dually stained with either 5 µg/ml DHE and 1 µg/ml Hoechst 33342 or 10 µg/ml EB [16] and 1 µg/ml Hoechst 33342. Hoechst 33342 is a cell membrane-permeable DNA stain [18] which was used as the comparator between DHE and EB; both of which are derived from the same parent molecule, ethidine and fluoresce red when bound to DNA. At 5 µg/ml, approximately 20% of the Hoechst-stained population (i.e. the iRBCs) was co-stained with EB (Figure 2A) whereas more than 90% erythrocytes in the Hoechst-stained population were co-stained with DHE (Figure 2B). This was also confirmed via confocal imaging (Figure 2C, D).

### Ratio of THP-1 Phagocytes to Erythrocytes in Phagocytic Assay

**Figure 3:**
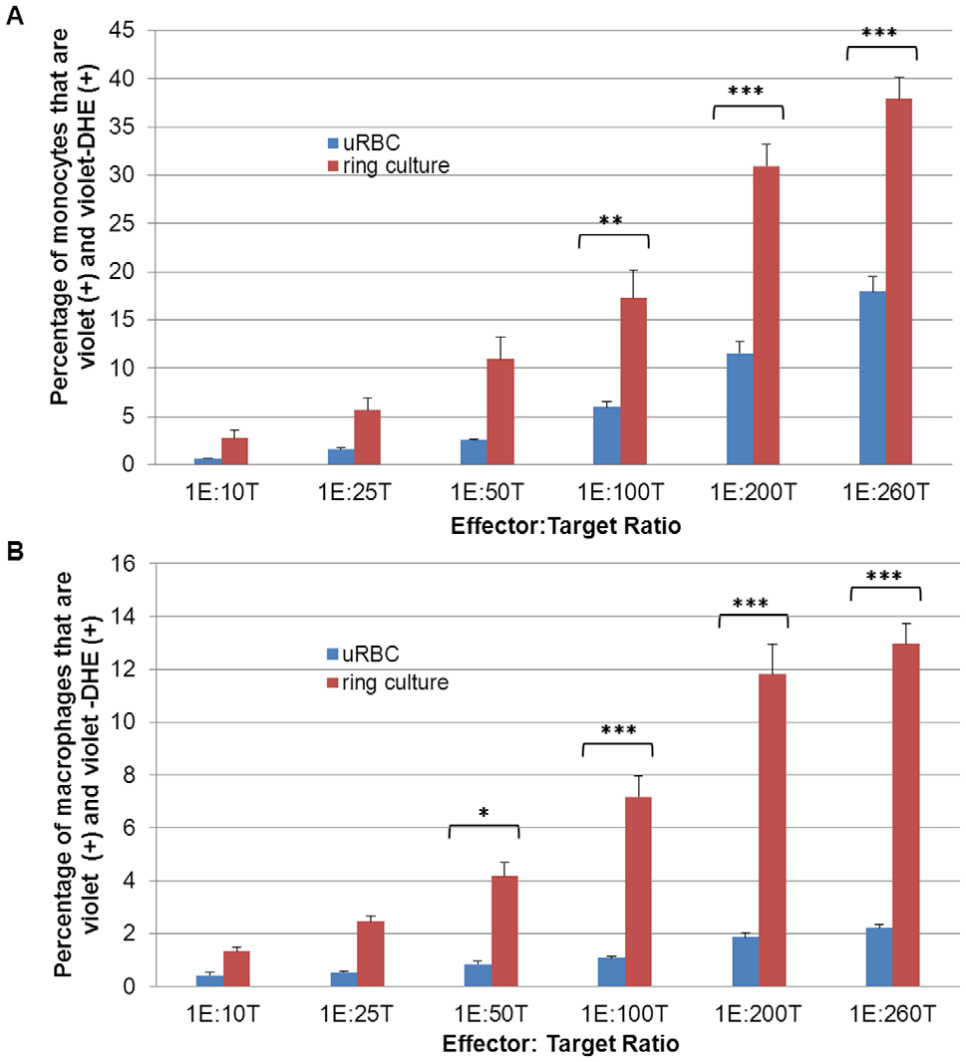
Optimisation of effector (THP-1) to target (erythrocytes) ratio. The effectors used in these experiments were (**A**) THP-1 monocytes, (**B**) PMA-differentiated THP-1 macrophages and the targets were fresh uninfected erythrocytes (uRBC) or ring-staged *Plasmodium falciparum* culture (ring culture) at 10% parasitemia. ____Percentage of effectors that have engulfed at least one erythrocyte (uninfected or infected) at effector : target (E:T) ratios from 1∶10 to 1∶260 after 4 h incubation at 37°C with pure effectors as the control. The experiments were done in duplicates at least 3 times with data expressed as mean ± SEM. Statistical analyses compared the number of effectors which had ingested erythrocytes from the uRBC culture with that from ring culture at the same E:T ratio. (*represents p<0.05, **represent p<0.005, ***represent p<0.001).

After confirming successful PMA differentiation via the up regulation of CD36 and CD68 in the macrophages (Figure S2), we optimized the effector to target (E:T) ratio in the phagocytic assay by incubating THP-1 effectors with varying numbers of erythrocyte targets (uninfected and infected) for 4 h at 5% CO_2_ in 37°C. There was an increase in erythrocyte uptake by the effectors with higher proportions of targets (Figure 3). In Figure 3A, with monocytes as effectors, the level of phagocytosis of infected ring-staged cultures was higher than that of uninfected ones at E:T ratios of 1∶100 (5.9±0.6% with uRBC and 17.2±2.9% with ring culture; p<0.005), 1∶200 (11.6±1.2% with uRBC and 31.0±2.2%; p<0.001)and 1∶260 (17.9±1.6% with uRBC and 37.9±2.2% with ring culture; p<0.001). From Figure 3B, the level of phagocytosis of infected ring-staged cultures by macrophages was significantly higher than that of uninfected ones at E:T ratios of 1∶50 (p<0.05) and 1∶100, 1∶200 and 1∶260 (all 3 with p<0.001). At 1E:50T, the total uptake by THP-1 macrophages of fresh uRBC was 0.8±0.1% compared to the uptake of ring cultures, 4.2±0.5%. Uptake of fresh uRBC was 1.1±0.1% compared to the uptake of ring cultures at 7.2±0.8% at1E:100T; 1.8±0.2% compared to 11.8±1.1% at 1E:200T and 2.2±0.1% compared to 13.0±0.8% at 1E:260T. An effector: target ratio of 1E:100T was selected for both monocytes and macrophages; the difference between the phagocytic levels of fresh uRBC and ring cultures were approximately 3-folds in monocytes (Figure 3A) and 6-folds in macrophages (Figure 3B).

### Flow Cytometry Analysis of Phagocytosis

With the establishment of the phagocytic assay using a ratio of 1E:100T, several control experiments were carried out to validate the effectiveness of this method in reporting phagocytic activity of phagocytes.

**Figure 4:**
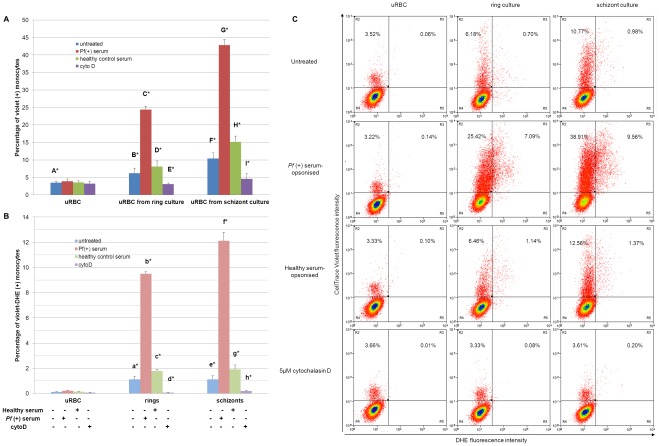
Phagocytosis of fresh uninfected erythrocytes (uRBC), ring-staged (ring culture) and schizont-staged cultures (schizont culture) at 10% parasitemia under different conditions by THP-1 monocytes. In standard conditions, incubation of erythrocytes with monocytes was carried out for 4 h at 37°C with an E:T ratio of 1∶100. To prevent phagocytosis, monocytes were pretreated with 5 µM cytochalasin D for 1 h before the phagocytic incubation of 4 h at 37°C. To increase phagocytosis, erythrocytes were opsonised with serum from a *Plasmodium falciparum* (+) patient at room temperature for 30 min before incubation with monocytes. A) Monocytes that have engulfed at least one uRBC and B) monocytes that have engulfed at least one iRBC. Data expressed as mean ± SEM. (B*vE*, p<0.05; a*vd*, p<0.005; A*vF*, C*vD*, F*vI*, G*vH*, b*vc*, f*vg*, p<0.001; e*vh*, p = 0.063; A*vB*, p = 0.097; n = 3 separate experiments, each in duplicates) C) Representative dotplots of the respective conditions when exposed to THP-1 monocytes.

**Figure 5:**
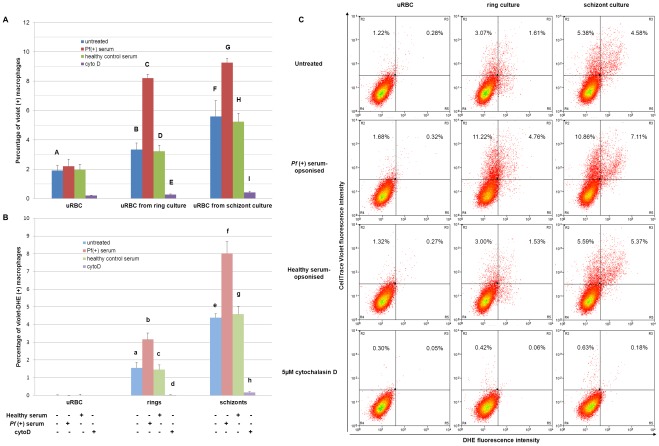
Phagocytosis of fresh uninfected erythrocytes (uRBC), ring-staged (ring culture) and schizont-staged cultures (schizont culture) at 10% parasitemia under different conditions by THP-1 differentiated macrophages. In standard conditions, incubation of erythrocytes with macrophages was carried out for 4 h at 37°C with an E:T ratio of 1∶100. To prevent phagocytosis, macrophages were pretreated with 5 µM cytochalasin D for 1 h before the phagocytic incubation of 4 h at 37°C. To increase phagocytosis, erythrocytes were opsonised with serum from a *Plasmodium falciparum* (+) patient at room temperature for 30 min before incubation with macrophages. A) Macrophages that have engulfed at least one uRBC and B) macrophages that have engulfed at least one iRBC. Data expressed as mean ± SEM. (bvc, p<0.05; BvE, p<0.005; AvF, CvD, FvI, GvH, evh, fvg, p<0.001; avd, p = 0.098; n = 3 separate experiments, each in duplicates) C) Representative dotplots of the respective conditions when exposed to THP-1 macrophages.

After incubation with phagocytes for 4 h, there was a significant increase in the uptake of uRBC in schizont cultures compared to fresh uninfected cultures. However, a similar comparison between uRBC in ring cultures and fresh uninfected cultures was not significant in both monocytes and macrophages. With monocytes, uptake of fresh uRBC was 3.5±0.4% compared to uRBC from schizont cultures (10.4±1.6%; p<0.001; Figure 4A). Fresh uRBC uptake by macrophages was 1.9±0.4% compared to uRBC from schizont cultures (5.6±1.1%; p<0.001; Figure 5A). We also noticed an increase in macrophage phagocytosis of schizont-staged iRBC (4.4±0.2%) compared to ring-staged iRBC (1.6±0.3%) with p<0.005 (Figure 5B) but not in monocytes (Figure 4B). The uptake of ring and schizont iRBCs in monocytes were similar to ring iRBC uptake in macrophages.

When the phagocytes were pretreated with cytochalasin D, a reversible actin polymerisation inhibitor [19], the level of phagocytosis was reduced in both iRBCs and uRBCs in *P. falciparum-*infected cultures (Figure 4A,B and Figure 5A,B). uRBC uptake in ring cultures by monocytes dropped from 6.2±1.4% when untreated to 3.1±0.3% with cytochalasin D treatment (p<0.05). In schizont cultures, uRBC uptake levels were reduced from 10.4±1.6% to 4.6±1.5% (p<0.001) after cytochalasin D treatment (Figure 4A). Looking at phagocytic levels in macrophages (Figure 5A), uRBC uptake in ring cultures decreased from 3.3±0.5% to 0.3±0.05% (p<0.005) and that in schizont cultures decreased from 5.6±1.1% to 0.4±0.1% (p<0.001). In addition, phagocytosis of ring-staged iRBC uptake by monocytes was inhibited (from 1.1±0.2% to 0.06±0.02%; p<0.005; Figure 4B). Schizont-staged iRBC uptake by macrophages reduced from 4.4±0.2% to 0.2±0.05%, (p<0.001; Figure 5B).

Phagocytic levels of *P. falciparum-*infected erythrocyte cultures increased significantly after opsonisation with heat-inactivated immune serum from a *P. falciparum*-positive patient (*P. falciparum* (+) serum). With monocytes, ring-staged iRBC uptake increased from 1.1±0.2% to 9.5±0.2% (p<0.001) and schizont-staged iRBC uptake from 1.1±0.3% to 12.1±0.7% (p<0.001) when comparing samples opsonised by healthy human serum or *P. falciparum* (+) serum respectively (Figure 4B). In a similar comparison (Figure 5B), phagocytosis by macrophages was also increased in ring-staged iRBC (from 1.5±0.3% to 3.2±0.4%; p<0.05) and schizont-staged iRBC (from 4.6±0.4% to 8.0±0.7%, p<0.001).

This increase in phagocytosis after *P. falciparum* (+) serum opsonisation was observed not only in iRBC uptake but also in uRBC uptake. With monocytes (Figure 4A), uRBC uptake in ring cultures increased from 8.1±1.6% to 24.4±1.0% (p<0.001) and uRBC uptake in schizont cultures from 15.2±1.6% to 42.9±1.6% (p<0.001) when comparing again samples opsonised by healthy human serum and *P. falciparum* (+) serum. The same increase was seen in macrophages (Figure 5A) with uRBC uptake in ring cultures increasing from 3.2±0.4% to 8.2±0.1% (p<0.001) and uRBC uptake in schizont cultures increasing from 5.6±1.1% to 9.3±0.3% (p<0.001).

**Figure 6:**
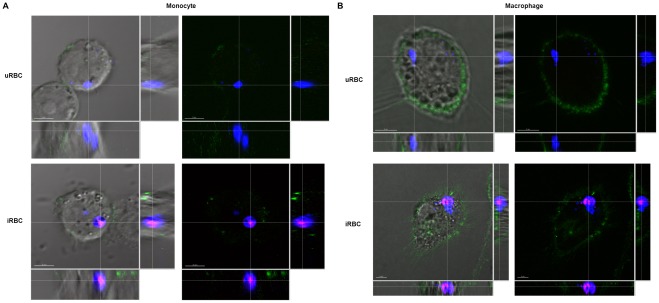
Confocal visualization of engulfed erythrocytes in monocytes and macrophages. Incubation of erythrocytes with phagocytes was carried out for 4 h at 37°C with an E:T ratio of 1∶100 and the phagocytes are subsequently labeled with FITC anti-human CD36 before viewing under the confocal microscope. Z-stack sections of **A**) a monocyte containing a CellTrace™ Violet- labeled uRBC or an iRBC labeled with both CellTrace™ Violet and DHE, **B**) a macrophage containing a CellTrace™ Violet- labeled uRBC or an iRBC labeled with both CellTrace™ Violet and DHE. (The scale bar represents 5 µm).

It was also interesting to note that a large proportion of the increase in phagocytosis observed for *P. falciparum*-infected cultures in the various experiments was attributed to uptake of uRBCs and not just iRBCs (Figure 4, 5), especially with monocytes. The monocytes showed a high uRBC uptake from infected cultures compared to the macrophages, particularly after *P. falciparum* (+) serum opsonisation. Samples were observed under confocal microscopy. Figure 6 shows 3D z-stack sections of phagocytes which have ingested either uRBCs or iRBCs from *P. falciparum-*infected cultures. FITC anti-human CD36 labeling the phagocyte surface demonstrated the erythrocytes were indeed ingested by the phagocytes. As expected, the level of FITC fluorescence was lower in monocytes than in macrophages, due to the abundant expression of CD36 in macrophages.

## Discussion

Phagocytosis of iRBCs and subsequent parasite antigen presentation is a crucial step in initiating the adaptive immune response for the eradication of the malaria infection. Understanding how antigen-presenting phagocytes interact with iRBCs could be important in discovering ways to make the transition from innate to adaptive immunity more efficient. The spleen is a major organ involved in the clearance of infected erythrocytes where damaged and infected erythrocytes are removed from the circulation via phagocytosis by splenic macrophages and dendritic cells. However, the majority of the later staged (trophozoite and schizont) iRBCs are sequestered in the microvascular endothelia of various organs [20] and thus ring-staged iRBCs are the main form in circulation, passing through the spleen periodically. Few studies have focused on ring-staged iRBCs, as opposed to the later more antigenic trophozoite and schizont stages. It is therefore relevant to study phagocytosis of the early ring-staged iRBCs, along with the later stages.

We described here a new labeling method using DHE and CellTrace™ Violet to label *P. falciparum*-infected cultures and visualize phagocytosis of erythrocytes, instead of EB and FITC, as previously reported [16]. This method enables researchers to assay the phagocytic levels of *P. falciparum* cultures at different developmental stages, particularly the ring stages, without the need for iRBC isolation.

iRBCs become more permeable in the course of *Plasmodium* development, with a marked increase in permeability during the trophozoite stage. This is due to the formation of new permeability pathways (NPPs) in the host cell membrane and it coincides with the enhanced metabolic activity of the parasites at this stage [21], [22]. DHE, a neutral molecule, is the chemically reduced form of EB. It is able to penetrate the intact membranes of host erythrocytes, even in the early ring stages, before it is oxidized to form positively-charged ethidium which intercalates with DNA in the parasite nucleus [23]. In contrast, EB is directly ionized into ethidium and bromide ions in solution. These charged particles can only pass through the membrane after the parasite develops and creates NPPs in the host erythrocyte membrane during the later trophozoite/schizont stages. Hoechst 33342 is another frequently used DNA stain and like DHE, it can label iRBCs of different stages as well. But Hoechst has poor cellular retention and diffused out of the iRBCs to stain phagocyte nuclei as well, making it difficult to differentiate by flow cytometry, whether an iRBC had been ingested (data not shown). In addition, detection of Hoechst by flow cytometry requires a UV laser. To date, this laser is only available in large and sophisticated instruments. Hence, DHE, which can be used with cheaper and commonly available 488 nm laser, is the ideal stain when labeling ring staged-iRBCs with relatively unmodified cell membranes.

To label the erythrocyte cytoplasm, we have employed CellTrace™ Violet which is excited at 405 nm and emits at 450 nm. The emission spectra of CellTrace™ Violet and DHE do not overlap; hence infected and uninfected cells can be distinguished easily without fluorescence bleed-through.From the results, we noted that there were high levels of phagocytosis of uRBC in parasite cultures. With the formation of NPPs and hence increased permeability in erythrocyte membrane, there could be release of parasite antigens such as ring surface protein 2 (RSP2) into the surroundings which might bind directly to neighboring uninfected cells [24], [25], [26]. It was also found that uRBCs cultured with *P. falciparum* displayed reduced deformability [27] and accelerated senescence in comparison to control uRBCs of the same age [28]. Experiments done in our laboratory have shown that uRBCs cultured with *P. falciparum* or incubated with spent culture media exhibit increased phosphatidylserine (PS) exposure on their cell surfaces (data not shown). Exposure of PS molecules which are normally confined to the inner leaflet of the cell membrane is a well-characterised apoptotic feature and it signifies an “eat-me” signal to phagocytes [29]. These, taken together, could result in an increase in detection by phagocytes and thus suggest reasons for the high levels of uRBC phagocytosis. These findings concur with studies demonstrating that majority of erythrocytes lost in falciparum malaria patients are uninfected [30], [31] and can explain why the extent of anemia does not always correlate with parasite density. Indeed, recognition and elimination of uRBC would lead to an accelerated development of anemia.

As the parasite develops, parasite antigens are inserted into the host erythrocyte membranes, particularly during the later stages of development. These neoantigens are easily recognised by phagocytes [5], hence explaining the increased uptake of iRBCs as they developed from rings to schizonts. With the addition of *P. falciparum* (+) serum for opsonisation, we saw a significant increase in phagocytic levels of the *P. falciparum*-infected iRBCs due to increased efficiency of Fc receptor (FcR)-mediated phagocytosis.

While papers have shown that macrophages generally display greater phagocytic ability than monocytes [32], [33], our study showed a different result. The trend held where phagocytic levels of unopsonized schizont iRBCs by macrophages were higher than those of unopsonized schizont iRBCs in monocytes, probably due to an increase in surface receptors such as CD36 [34], [35]. But it is presently unclear why monocyte phagocytic levels of uRBCs in infected cultures were elevated, particularly after *P. falciparum* (+) serum opsonization. More studies investigating this phenomenon are warranted.This method was able to demonstrate phagocytic increase via serum opsonisation and inhibition by cytochalasin D, and was sensitive enough to detect differences in uptake between uninfected erythrocytes and various stages of iRBCs. It was also relatively quick as staining with CellTrace™ Violet and DHE was done concurrently in 20 minutes. In this study, we focused on monocytes and macrophages as the principle phagocytes but this method could be extended for use with other phagocytes such as dendritic cells or neutrophils for the study of parasite-phagocyte interactions.

Finally, the method also can be used to compare the phagocytic capacities of different monocyte/macrophage populations. Different monocyte/macrophage subsets exhibit different functions [36], [37] and thus might differ in their capacity to phagocytosis iRBCs or uRBCs which have been in contact with the parasite. In addition, the influence of genetic polymorphisms for proteins involved in phagocytosis can be investigated using this assay, in particular, those known to be associated with protection from severe malaria (for example CD36 and FcRs) [38], [39], [40]. Lastly, it would be interesting to compare the phagocytic efficiency of one particular monocyte/macrophage subset for different erythrocytes infected with different *Plasmodium* strains or field isolates and expressing different sets of neo-antigens, in the presence or absence of immune sera.

## Materials and Methods

### Ethics

The blood collection protocol for malaria in vitro culture was approved by the Institutional Review Board (IRB) (NUS-IRB Ref Code: 09-141, Approval Number: NUS-782) of the National University of Singapore (NUS). Written informed consent was obtained from all participants involved in the study.

### Parasite Culture and Erythrocyte Labeling

*Plasmodium falciparum,* strain 3D7 (MRA-102, MR4, ATCC, Manassas, VA, USA) *in vitro* cultures were maintained in 75 cm^2^ flasks with human erythrocytes (blood group O^+^, erythrocytes with less than 2 weeks of storage at 4°C) in malaria culture media (MCM), which consisted of RPMI 1640 medium supplemented with 0.5% (w/v) Albumax II (Invitrogen, Auckland, New Zealand), 2 mM L-Glutamine, 0.3 mM hypoxanthine, 25 µg/ml gentamycin, at 1% hematocrit. Subculturing was done on alternate days; the cultures were gassed with 5% CO_2_ (v/v) and 3% O_2_ (v/v) balanced with N_2_ and kept at 37°C in a dark incubator. Synchronization was done twice weekly using the 5% D-sorbitol lysis method, to obtain tightly synchronized cultures before use in experiments. Thin blood smears stained with Giemsa were used to determine parasite developmental stage and parasitemia, before subculturing and prior to each experiment. Parasite cultures of approximately 10 to 20% parasitemia were used in all experiments.

To optimize the DHE concentration necessary to clearly visualize the parasites, synchronized *P. falciparum* infected ring-staged cultures were stained with various concentrations of DHE (5 to 50 µg/ml; Molecular Probes, Invitrogen, Eugene, Oregon) for 20 mins at 37°C. In other experiments, ring cultures were stained with EB or DHE in combination with Hoechst 33342 (Molecular Probes, Invitrogen) for 20 mins at 37°C. Labeled erythrocytes were washed with PBS twice before analysis by flow cytometry (gating of which is shown in Figure S3) and visualization under confocal microscopy.

For phagocytic experiments, ring-staged cultures were centrifuged at 800 g for 3 mins to pellet the erythrocytes. The supernatant was removed and the pellet was resuspended in PBS before staining with DHE and 2 µM CellTrace™ Violet (Molecular Probes, Invitrogen) for 20 mins at 37°C. Labeled erythrocytes were washed twice with 10 volumes of fresh MCM and finally resuspended in MCM before incubation with phagocytes.

### Monocyte Culture and Macrophage Differentiation

Non-adherent human monocyte cell line THP-1 (provided by Dr Sylvie Alonso, Immunology Programme, NUS) was maintained in 150 cm^2^ flasks with RPMI 1640 medium supplemented with 10% (v/v) fetal bovine serum (Gibco, Grand Island, NY), 2 mM L-Glutamine, 100 units/ml penicillin and 100 ug/ml streptomycin (THP-1 culture medium). The cells were subcultured every 3 days and density was maintained at less than 2×10^5^ cells per ml; cultures were kept in a humidified 37°C incubator with 5% (v/v) CO_2_ and 95% (v/v) air. A viable count was done on THP-1 cells using a haemocytometer and trypan blue prior to experiments.

THP-1 cells were seeded at 5×10^5^ cells per well in 12-well plates (Greiner Cellstar, Frickenhausen, Germany) and the volume of each well was made to 3 ml with THP-1 culture media. To obtain macrophages, the cells were differentiated using 10 ng/ml phorbol 12*-*myristate 13*-*acetate (PMA; Sigma-Aldrich, Dorset, UK) for 24 h in 5% (v/v) CO_2_ at 37°C. The supernatant and unattached cells were removed by aspiration and adherent macrophages were washed twice with THP-1 culture medium before the wells were filled with 3 ml of fresh THP-1 culture medium. These were incubated a further 48 h before use for phagocytic experiments.

Expression of cell surface markers, CD36 and CD68, were compared between THP-1 monocytes and differentiated macrophages to ensure successful differentiation. The differentiated cells were washed twice with PBS and incubated with warmed cell-lifting reagent (PBS with 5 mM EDTA and 10 mM D-glucose, pH 7.2) for 10 min at 37°C before being detached gently with a cell scraper. Both monocytes and differentiated macrophages were washed, resuspended in 200 µl of PBS and incubated with APC-Cy7-conjugated antihuman CD36 and PE-conjugated antihuman CD68 (provided by Dr Wong Siew Cheng, Singapore Immunology Network, A^*^STAR) for 30 mins at 4°C. They were washed trice with PBS and resuspended in 500 µl of PBS before flow cytometric analysis.

### Phagocytosis by THP-1 Differentiated Macrophages

Fresh uninfected erythrocytes or *P. falciparum* infected ring-staged cultures (ring culture) labeled with DHE and CellTrace™ Violet were added to wells containing THP-1 monocytes and macrophages at varying effector (E) to target (T) ratios (from 1E:10T to 1E:260T), where the effectors were THP-1 cells and the targets were erythrocyte cultures. The cells were incubated for 4 h at 37°C in 5% (v/v) CO_2_. Adherent THP-1 macrophages were washed with PBS twice to remove unphagocytosed erythrocytes. The cells are then treated with 500 µl 0.25% trypsin-EDTA for 5 mins at 37°C to detach them and washed in 2 volumes of THP-1 culture medium. For the monocytes, the cells were resuspended in 1 ml red cell lysis buffer (distilled water with 1.7 mM Tris, 0.14 M ammonium chloride at pH 7.4) at 37°C for 8 mins with frequent agitation, to lyse the unphagocytosed erythrocytes before being washed twice with 10 volumes of THP-1 culture media. After which, both monocytes and macrophages were then resuspended in 500 µl PBS for flow cytometric analysis. E:T ratio of 1∶100 was chosen for subsequent experiments.

In experiments to validate the method, THP-1 monocytes and differentiated macrophages were incubated with DHE- and CellTrace™ Violet-labeled fresh uninfected erythrocytes, ring cultures and schizont cultures under various conditions. To inhibit phagocytosis, 1) THP-1 phagocytes were preincubated with 5 µM cytochalasin D (Sigma-Aldrich), an actin polymerisation inhibitor, for 1 h at 37°C prior to the addition of labeled erythrocytes for phagocytosis. To increase phagocytosis, 2) labeled erythrocytes were opsonised with heat-inactivated immune serum (*P. falciparum* (+) serum) from a *P. falciparum*-positive patient (a kind gift of Prof Francois Nosten, Shoklo Malaria Research Unit, Mae Sod, Tak Province, Thailand) for 30 mins at room temperature and washed thrice before being added to THP-1 phagocytes for phagocytosis. As a control, labeled erythrocytes were also opsonized with heat-inactivated healthy AB human serum (Life Technologies Inc.) in the same manner.

### Flow Cytometry and Confocal Microscopy

For flow cytometry, samples were acquired with CyAn ADP Analyzer (Dako/Beckman Coulter, Brea, CA, USA), fitted with 405 nm and 488 nm solid state lasers, and analyzed using the Summit software, version 4.3. The THP-1 effector cells were selected according to their forward and side scatter properties using THP-1 cells alone as a control. Selected events were displayed on violet (FL 6) fluorescence versus red (FL 3) fluorescence dotplots. THP-1 cells containing phagocytosed CellTrace™ Violet-labeled uRBCs were defined by a region set for violet fluorescence and those containing iRBCs (labeled with both CellTrace™ Violet- and DHE) were defined by a region set for dual-positive violet and red fluorescence. At least 20,000 events were collected for each phagocytic sample. After phagocytic incubation, effector cells were labeled with FITC anti-human CD36 (Biolegend, CA, USA) before confocal imaging. Confocal imaging was performed under 100× oil objectives using Olympus Fluoview FV1000 (Tokyo, Japan) with the software Olympus Fluoview version 2.0A and 3D reconstruction was done using Imaris ×64 version 6.1.5.

### Statistical Analyses

Statistical significance of differences between the experimental groups as indicated was analyzed by ANOVA with post-hoc comparison using Tukey’s test for paired comparisons. Significantly different results (p<0.05) were highlighted. All statistical analyses were performed using PASW Statistics 18, Release 18.0.0.

## Supporting Information

Figure S1 Giemsa stain of ring-staged 3D7 *Plasmodium falciparum* cultures with a parasitemia of about 15%.
(TIF)

Figure S2 **Expression of surface markers on THP-1 cells before and after PMA differentiation.** THP-1 monocytes (solid black line) and THP-1 macrophages differentiated with 10 ng/ml PMA (solid gray line) were incubated with **A**) APC-Cy7 antihuman CD36 and **B**) PE antihuman CD68 for 30 min at 4°C. The differentiated macrophages showed an up-regulation of CD36 and CD68 compared to the monocytes.
(TIF)

Figure S3 **Forward and side scatter plot of 3D7** ***Plasmodium falciparum*** **cultures with the R18 gating used to analyze the erythrocyte population for determining optimal DHE concentration.**
(TIF)
