# Supplementary figures and images for: A Simplified, Sensitive Phagocytic Assay for Malaria Cultures Facilitated by Flow Cytometry of Differentially-Stained Cell Populations
Source: PLoS One. 2012 Jun 4;7(6):e38523. doi: 10.1371/journal.pone.0038523 (PMC3366917; doi:10.1371/journal.pone.0038523)

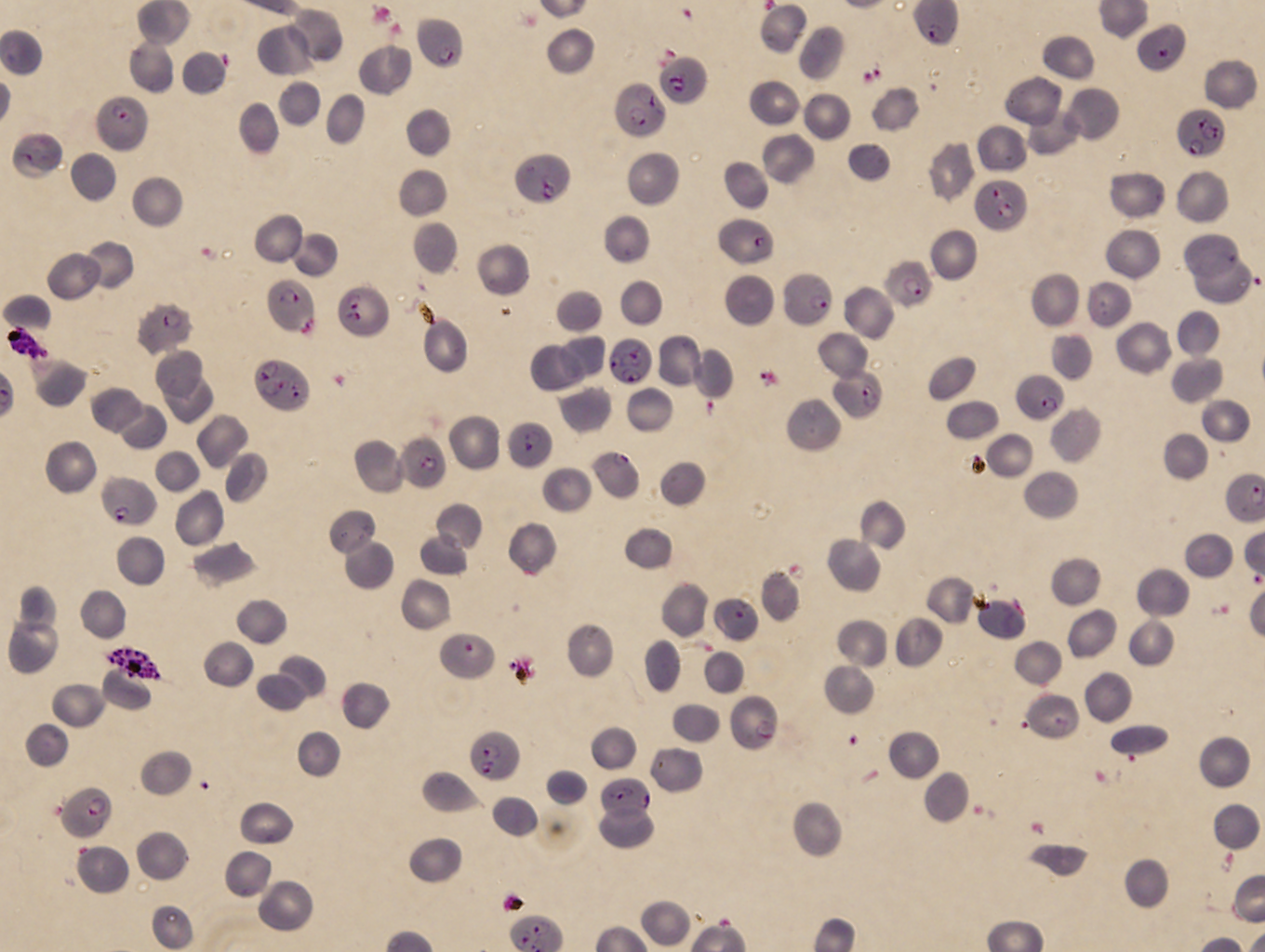

Supplement: Figure S1 — Giemsa stain of ring-staged 3D7 Plasmodium falciparum cultures with a parasitemia of about 15%. (TIF) [file pone.0038523.s001.tif]

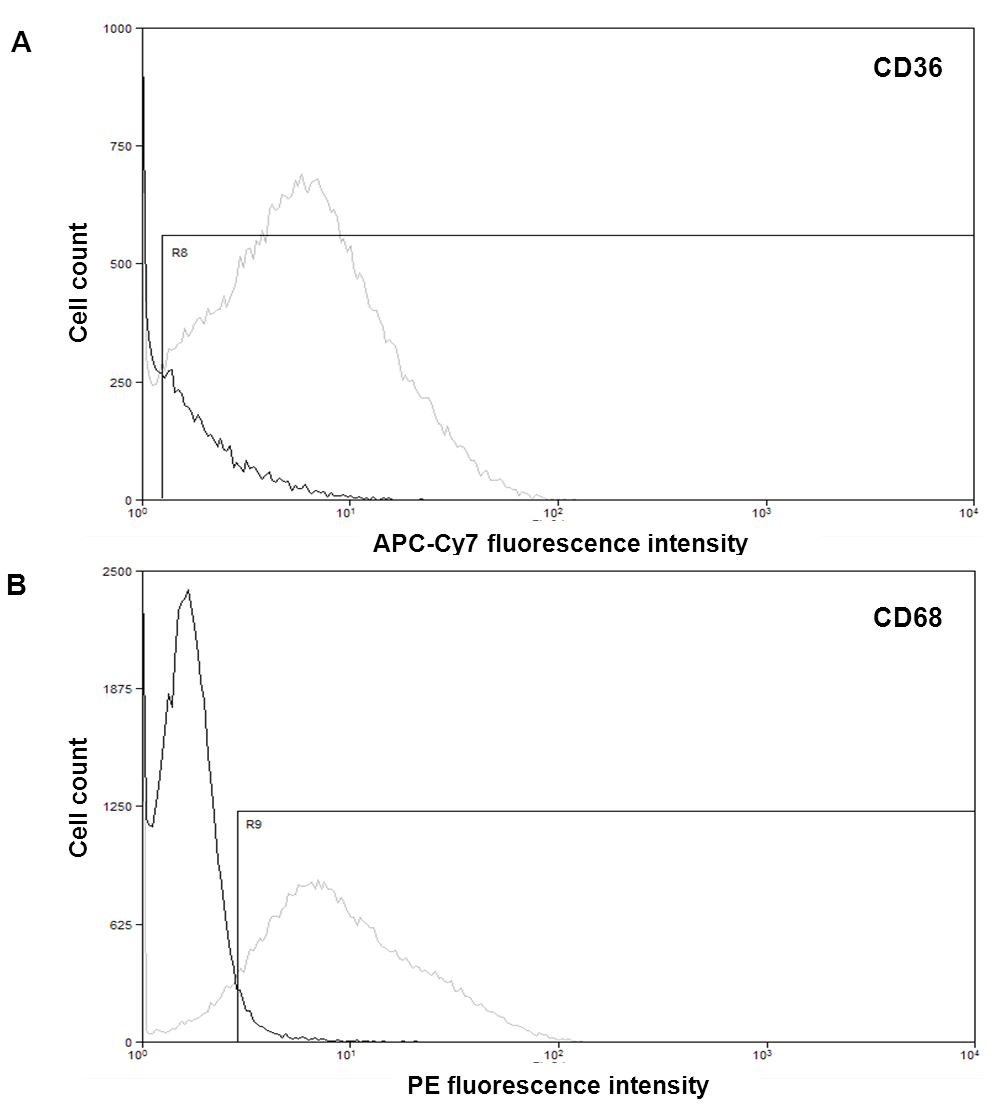

Supplement: Figure S2 — Expression of surface markers on THP-1 cells before and after PMA differentiation. THP-1 monocytes (solid black line) and THP-1 macrophages differentiated with 10 ng/ml PMA (solid gray line) were incubated with A) APC-Cy7 antihuman CD36 and B) PE antihuman CD68 for 30 min at 4°C. The differentiated macrophages showed an up-regulation of CD36 and CD68 compared to the monocytes. (TIF) [file pone.0038523.s002.tif]

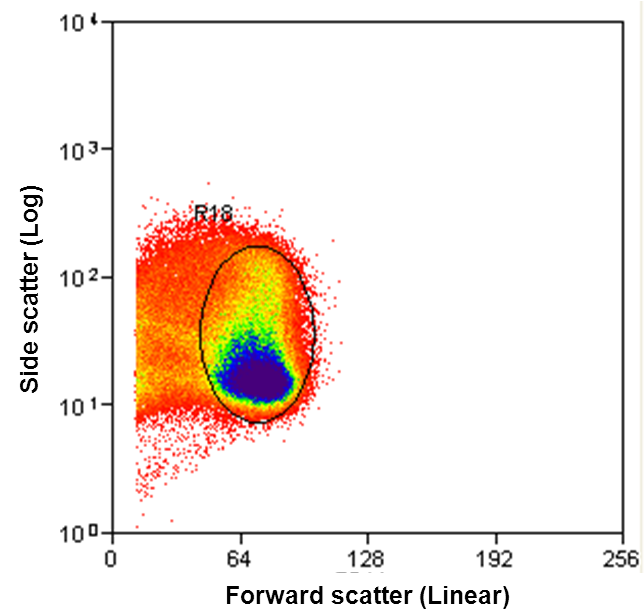

Supplement: Figure S3 — Forward and side scatter plot of 3D7 Plasmodium falciparum cultures with the R18 gating used to analyze the erythrocyte population for determining optimal DHE concentration. (TIF) [file pone.0038523.s003.tif]
